# The Cuban Human Brain Mapping Project, a young and middle age population-based EEG, MRI, and cognition dataset

**DOI:** 10.1038/s41597-021-00829-7

**Published:** 2021-02-05

**Authors:** Pedro A. Valdes-Sosa, Lidice Galan-Garcia, Jorge Bosch-Bayard, Maria L. Bringas-Vega, Eduardo Aubert-Vazquez, Iris Rodriguez-Gil, Samir Das, Cecile Madjar, Trinidad Virues-Alba, Zia Mohades, Leigh C. MacIntyre, Christine Rogers, Shawn Brown, Lourdes Valdes-Urrutia, Alan C. Evans, Mitchell J. Valdes-Sosa

**Affiliations:** 1 The Clinical Hospital of Chengdu Brain Sciences, University of Electronic Sciences and Technology of China, Chengdu, China; 2 Cuban Neuroscience Center, La Habana, Cuba; 3 McGill Centre for Integrative Neurosciences MCIN. Ludmer Centre for Mental Health. Montreal Neurological Institute, McGill University, Montreal, Canada

**Keywords:** Electroencephalography - EEG, Magnetic resonance imaging, Diagnostic markers, Brain imaging, Biophysical models

## Abstract

The Cuban Human Brain Mapping Project (CHBMP) repository is an open multimodal neuroimaging and cognitive dataset from 282 young and middle age healthy participants (31.9 ± 9.3 years, age range 18–68 years). This dataset was acquired from 2004 to 2008 as a subset of a larger stratified random sample of 2,019 participants from La Lisa municipality in La Habana, Cuba. The exclusion criteria included the presence of disease or brain dysfunctions. Participant data that is being shared comprises i) high-density (64–120 channels) resting-state electroencephalograms (EEG), ii) magnetic resonance images (MRI), iii) psychological tests (MMSE, WAIS-III, computerized go-no go reaction time), as well as iv,) demographic information (age, gender, education, ethnicity, handedness, and weight). The EEG data contains recordings with at least 30 minutes in duration including the following conditions: eyes closed, eyes open, hyperventilation, and subsequent recovery. The MRI consists of anatomical T1 as well as diffusion-weighted (DWI) images acquired on a 1.5 Tesla system. The dataset presented here is hosted by Synapse.org and available at https://chbmp-open.loris.ca.

## Abstract (summary)

**Table:** 

Measurement(s)	functional brain measurement
Technology Type(s)	electroencephalography (EEG) • magnetic resonance imaging (MRI) • neuropsychological testing
Factor Type(s)	age of participants • gender of participants • handedness of participants • educational level of participants
Sample Characteristic - Organism	Homo sapiens
Sample Characteristic - Location	Cuba

Machine-accessible metadata file describing the reported data: 10.6084/m9.figshare.13277348

## Background & Summary

In the past decade several neuroimaging databases (ADNI, HCP, UK Biobank, CAMCAN, ABCD, PPMI), as well as consortia (ENIGMA), have been launched. They aim to accelerate insights into neurodevelopment and physiopathology and to allow the identification of new biomarkers. An essential ingredient, lacking in many projects, is the inclusion of the electroencephalograms (EEG), an informative and direct measurement of brain activity. The EEG is cost-effective, accessible, and applicable to underserved populations throughout the world. It is a technique of choice for extensive population screening in any economic setting. It is not surprising, therefore, that interest in EEG has increased, and this modality has been included in new multimodal neuroimaging datasets, such as the mind-brain-body dataset CMI (Babayan *et al*.^1^) and the open resource for transdiagnostic research in pediatric mental health LEMON (Alexander *et al*.)^1,2^.

Here we present a new multimodal neuroimaging dataset^3^ that includes, this time information from a Latin American middle-income country. It was led by the Cuban Human Brain Mapping Project (CHBMP)^4^, a population-based, multi-decade longitudinal brain health data-gathering effort in Havana Cuba. This ongoing project is organized by the Cuban Ministry of Public Health (MINSAP) and coordinated by the Cuban Neuroscience Center (CNEURO). The CHBMP focuses on the development of tools and health applications based on multimodal neuroimaging. This data corresponds to a young to middle-age adult population. Due to an official request from the Ministry of Health of Cuba (for a special study) the sampling of males according to the randomized sampling plan continued longer than that of females, resulting in a smaller number of the latter. This gender imbalance will be corrected in ongoing studies. Also two subsequents studies comprising childhood and elderly are in progress to answer scientific questions related to neurodevelopment and healthy aging. The results of these studies will be reported in due course.

The CHBMP has focused on quantitative evaluation of the EEG or “qEEG”^5^. qEEG helps identify brain disorders and is therefore a potentially valuable screening tool. In qEEG, the scalp recorded EEG log spectra of a proband is compared with normative log spectra using statistical parametric mapping procedure. These normative log spectra are age-dependent means and standard deviations, obtained by analyzing a large sample of healthy participants over a wide age range. An EEG normative database is thus a prerequisite for qEEG.

The need for a Cuban normative database for qEEG^6^ thus prompted the first wave of the CHBMP, initiated in 1988. It included 211 healthy persons (ages 5 to 97 years)^7^. Participants were randomly selected from the population and screened by the Family Doctor system to include only healthy participants. This database was used to develop a high-resolution qEEG validated by the Cuban Health system^8,9^. It also prompted the development of qEEG for sources (qEEGt). Due to the unavailability of MRI in Cuba at this time, an “approximate qEEGt” was based on the average head model developed by the ICBM consortium^10,11^. Procedures to apply qEEG and qEEGt processing based on this dataset have been integrated into CBRAIN^12^.

However, average-brain template-based qEEGt does not deal adequately with individual cortical anatomy, Thus qEEGt based on each individual’s MRI, as in^13^, is important to validate approximate qEEG but also for integrative multimodal neuroimaging studies. This motivated the second wave of the CHBMP (from 2004 to 2008) as one of the projects for the National Program for Disability.

As in the first wave of the CHBMP, the participants were recruited from the general population using stratified randomized sampling. This yielded 2,019 candidates who were screened, Family Nurses and Doctors, and extensive clinical, neurological, psychological, and neuroimaging evaluations to exclude participants with brain disorders, addictive habits, or exclusionary health conditions, resulting in a final sample of 282 “functionally healthy” participants. The recording protocol included high-resolution EEG, T1, MRI, DWI, and psychological tests such as MMSE, Wechsler Adult Intelligence Scale (WAIS-III), computerized reaction time, as well as the collection of blood samples for a genome-wide association study (GWAS) to be described in a separate further publication.

Making this dataset open is part of the Cuba-Canada-China (CCC) and the Global Brain Consortium (GBC) strategy to integrate EEG neuroimaging as an essential component of multimodal Neuroimaging, and to serve as a “translational bridge” for resource-limited scenarios. This is possible by integrating CHBMP efforts into the MNI neuroinformatics ecosystem^14^, based on the CBRAIN processing portal^15^ for the processing modules and the use of the LORIS database system for data storage and open access.

## Methods

### Participants

The Cuban Human Brain Mapping Project second-wave database contains neuroimaging, medical and cognitive data from, 282 “functionally healthy” participants from the general Cuban population between ages 18–68, (31.9 ± 9.30), comprising 87 (36.5 ± 10.43) females and 195 (29.9 ± 7.97) males. Details of the number of participants in each interval of age is presented in Table 1.

**Table 1:** Distribution of participants per age interval.

Age Interval	Number of participants	%
**10 < x < = 20**	34	12.05
**20 < x < = 30**	90	31.91
**30 < x < = 40**	116	41.13
**40 < x < = 50**	29	10.28
**50 < x < = 60**	10	3.54
**60 < x < = 70**	3	1.06

Details of the demographically variable frequency of gender, self-referenced handedness, and educational level are presented in Table 2, and stored in the file ***Handedness.csv*** and ***Demographic_data.csv***, which also included the weight (lb) of the participants.

**Table 2:** Demographic description of the sample.

	Categories	N (%)
Gender	Female	87 (30.85%)
	Male	195 (69.14%)
Handedness by preference	Right	238 (84.39%)
	Left	26 (9.21%)
	Ambidextrous	8 (2.38%)
	*Missing*	10 (3.54%)
Education level	Primary school	16 (5.67%)
	Secondary School	72 (25.53%)
	High school	145 (51.41%)
	University	39 (13.82%)
	*Missing*	10 (3.54%)

### Ethics

This study was carried out following the Declaration of Helsinki^16^. The experimental protocols were approved by the Ethical Review Committee of the Cuban Neuroscience Center using the guidelines of the Public Health (MINSAP from Spanish) and the Science, Technology, and Environment Ministries of the Republic of Cuba (CITMA from Spanish). Participants included in the study signed a written consent after a house visit by the Primary Health care nurse who gave a verbal and written explanation of the purpose, risks, and benefits of the study. Participants also were informed about the confidentiality of their personal information as well as full access to the best diagnostic and therapeutic procedures in case any kind of illness were detected that might prevent them to form part of the normative dataset. Additionally, they were verbally informed about their right to obtain any clinical, psychological, and neuroimaging results. Finally, participants were informed about further publications which would result from the project, with the guarantee of anonymization and control of the privacy of their personal information.

### Recruitment and exclusion criteria

The recruitment procedure is summarized in Table 3 and subsequently detailed.

**Table 3:** General procedure for recruitment.

	Activity	Sample size
Stage 1	Selection of one municipality with the closest match in gender, age, ethnic characteristics of the general Cuban population. This was also a municipality with a large proportion of persons originally from other provinces according to the MINSAP and Statistics National Office. La Lisa municipality was selected. A random sample of 30,000 participants from the total population was selected based on the ID card serial numbers.	30,000
Stage 2	From the 30,000 participants selected in the previous stage, a random subsample of 2019 was selected stratified by age, sex, ethnic origin, educational level, and socioeconomic status.	2019
Stage 3	The Doctor and nurses of the Family Program evaluated the willingness to participate and any health conditions of the candidates from the subsample (N = 2019). In detail: 1) The Family nurses visited each household to provide both printed and verbal information about the project objectives and enquired about the preliminary willingness to participate in the study. 2) Those willing to participate attended an interview at their local Polyclinic center for further explanation and signed the Informed Consent. 3) The Health Questionnaire was then applied by the nurse to carry out a further screening of pathology, cognitive complaints, use of pharmaceutical agents, heavy smoking, etc. 4) According to the exclusion criteria, Table 3, a further selection of the subsample was carried out for the next stage. Participants with health problems remained in the overall study, and continue to be followed up to this day with further clinical evaluation and treatment. However, they were excluded from the normative part of the project.	1439
Stage 4	At the local Polyclinic center psychiatrist/neurologist applied: • A standardized and structured psychiatry tool, the MINI International Interview (MINI) • MINIMENTAL State Evaluation (MMSE) • The Edinburg Handedness Questionnaire to classify dexterity A psychometrist applied the: • The Weschler Adult Intelligence Scale (WAIS) III	530
Stage 5	At the local Polyclinic center, blood samples for genetic studies were extracted and additional measures: the blood pressure, body temperature, frequency and heart rate, body weight and height were obtained (data not included in this version of the database). The weight in pounds was included in demographic file. Digital EEG recording was the final step at the designated Policlinics	530
Stage 6	MRI collection was carried out at the CIMEQ Hospital followed by an MRI evaluation by a neuroradiologist. Due to a request of the Ministry of Health the number of males selected at this stage was more than double of females.	394

**Stage 1:** In agreement with the Ministry of Public Health (MINSAP), and due to logistical constraints, a single municipality of the province of Havana City was selected for the study, together with the whole structure of the Family Doctor and Polyclinic Centers. Towards this end, a study was carried out with a committee from MINSAP and the National Office for Population Studies to assess the distribution of the following variables of all inhabitants in every municipality in the Province of Habana City: ethnicity, sex, province of origin, and socioeconomic status. Based on these distributions, the Municipality of La Lisa, was selected for the study since it had the closest match to the general Cuban population. A sample of 30,000 inhabitants in this region was randomly selected from the National Identity Card registry.

**Stage 2:** From the original roster of 30,000, a random subsample of N = 2019 was then selected for further processing, is stratified by age, gender, socio-economic status.

Family Doctors (**Stage 3**) then examined the participant’s records to exclude persons whom they already had ascertained to have health issues. All the remaining participants were visited by the Family nurses who left a printed description of the project and gave a detailed verbal explanation of its aims. As usual for population studies in Cuba, it was explained that there would be no payment for the study, but if a participant needed to be absent from the workplace, the local government guaranteed this as a fully paid day. They were also informed about all data acquisition protocols as well as safety measures with a special focus on MRI acquisition and safety. To a great degree, the success of this project was due to the close contact of the Family Doctor and Nurse with the local population, as well as the abundant information provided from the media to the general public about the Cuban Neuroscience Center and its project. This explains a 93% initial willingness to participate in the project. For those participants that gave written consent, a health questionnaire was applied for further screening, and consequently, 580 persons were excluded at this stage from the normative study. In this, as well as in subsequent stages, all participants that did not continue in the normative study followed a separate workflow to ensure specialized diagnostic and intervention by units of the health system, with the same protocol as those continuing in the study. The exclusion criteria used for this stage are listed in Table 4. The most prevalent health conditions to exclude participants were diagnosed with metabolic syndrome, psychiatric conditions, personal history of severe illnesses, and sensory and motor disabilities.

**Table 4:** Exclusion Criteria.

Criteria	Description
Medical conditions	Malignant systemic disease requiring chemotherapy, diabetes, thyroid dysfunction (hyper or hypothyroidism), rheumatic disease, muscular dystrophies, liver cirrhosis, sickle cell anemia, Wilson’s disease, lupus erythematosus (SLE), malnutrition, cardiovascular diseases, arterial hypertension, infectious diseases, AIDS, respiratory diseases, history of reactions to medications with hypersensitivity type I, current pregnancy or lactation, permanent metal appliances, cardiac pacemakers or other types of metal in any part of the body (fixed prosthesis, fragments of a bullet, etc.)
Neurological disease	Chronic systemic diseases of the Central Nervous System (CNS), seizures or other attacks, malignant expansive processes of the SNC-radiotherapy, more than one loss of consciousness, cerebrovascular accidents, transient cerebral ischemia, migraine or frequent headaches, severe neuropathies, history of cranial trauma with or without loss of consciousness.
Psychiatric Disorder	History of previous psychiatric treatments, tics, stuttering, intellectual disability, suicide attempt, depression, sociopathic behavior, anxiety disorders, panic attacks, schizophrenia, obsessive-compulsive disorder, drug use and abuse, alcoholism, hyperactivity and attention deficit, dementia.
Prenatal and perinatal antecedents	Difficult pregnancy as indicated by the obstetrician, preterm delivery, gestational hypertension, diabetes, obesity, early membrane rupture in pregnancy, placenta previa, retroplacental hematoma, other infections.
Sleep disorders	Nocturnal terrors, somnambulism, and others.
Familiar pathological background	Epilepsy, neurodegenerative diseases, multiple sclerosis, Wilson’s disease, schizophrenia, manic depressive disorder.
Drug addiction	Alcohol abuse according to DSM-IV definition, non-legal drugs, smoker of more than 12 cigarettes per day, drugs affecting the CNS, direct and chronic exposure to toxic substances such as pesticides, heavy metals, phosphorus, and organic solvents.
Neurological physical examination	Any abnormality in the neurological physical examination (hypertonia, hypotonia, asymmetry of reflexes, decrease in visual acuity, nystagmus, etc.).

In **Stage 4**, participants continuing in the study were examined at the polyclinic by specialists in Neurology and Psychiatry, to rule out chronic diseases (e.g. addictions, including heavy smoking) or any disorders of the nervous system that would invalidate their participation. Neurological examination was performed following the procedure described in the guidelines published by the U.S. Department of Health and Human Services in 1997^17^ and the Mini-Mental State Examination^18^ (MMSE) for global cognitive screening. The Mini-International Psychiatric Interview was used for psychiatric evaluation^19^ (Spanish version) and the Weschler Adult Intelligence Scale (WAIS) III for intelligence.

In **Stage 5**, EEG recordings were carried out at the polyclinic. Other measures were obtained the same day such as anthropometric (height) and blood pressure. Additional blood samples were extracted, following a join protocol from CNEURO with the National Center for Medical Genetics https://www.ecured.cu/Centro_Nacional_de_Gen%C3%A9tica_M%C3%A9dica for further genetic studies of the Cuban population (to be published separately).

During stages 4 and 5 specific exclusion criteria were employed (see Table 5).

**Table 5:** The exclusion criteria according to cognitive, EEG, and MRI examination.

Cognitive	WAIS below 70 MMSE below 24
Visual EEG	Abnormalities in the background EEG activity characterized by any of the following: a) out of range average amplitude or dominant frequency, b) absence of normal modulation patterns, c) inadequate anterior/posterior organization according to age, d) paroxysms, e) lack of reactivity to eye-opening and/or hyperventilation procedures.
MRI	Neuroradiological reports of lesions or atypical findings.

In **Stage 6**, the MRI studies were carried out at the Center for Medical and Surgical Research la Habana, Cuba (in Spanish: Centro Investigaciones Medico-Quirurgicas, CIMEQ https://www.ecured.cu/Centro_de_Investigaciones_M%C3%A9dico_Quir%C3%BArgicas_

### (CIMEQ)

Additionally, a psychiatrist/psychometrist applied the computerized Reaction Time test at the end of the study for a subsample (N = 56).

The participants who presented hypertension during this research study were included in a separate study and underwent more specific evaluations such as carotid flow, white matter hyperintensities, eye fund, optic and blood vessel impairments, and a set of extra measurements. The analysis of the results of this hypertension study was partially published in^4^.

#### Procedure

##### Workflow.

**Fig. 1:**
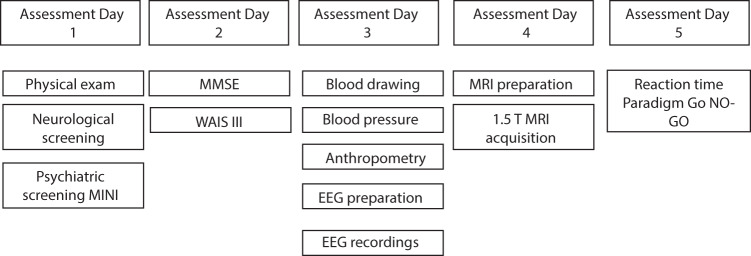
Flow-chart of 5 days assessment.

Examinations for final participants (Stages 4–6) were carried out in a five-day schedule. See Fig. 1.

**T**he final sample count for this study (N = 282) included all the participants who completed all the requisites, after all the steps. The total participants included in each measurement and the conjunction between modalities can be found in Table 6.

**Table 6:** Number of participants with each type of measurement.

Measurement	Number
EEG	250. 170 participants with 64 and 80 with 120 channels.
MRI	203
WAIS	167
MMSE	156
RT	56
EEG + MRI	171
EEG + MRI + WAIS	142
EEG + MRI + MMSE	132

### Psychological tests

#### MMSE

The Mini-Mental State Examination MMSE^18^ is a quick and easy measure of cognitive functioning that has been widely used in clinical evaluation and research involving patients with dementia. In our study, the MMSE was employed as a screening test to exclude participants with cognitive impairment. The total score of the participants is available in the file ***MMSE.csv*** with also the individual items for 52 subjects.

#### Intelligence test

Intelligence was assessed using the fully validated and translated to the Spanish language version of the David Wechsler Adult Intelligence Scale WAIS-III printed and distributed in Mexico by El Manual Moderno (https://www.worldcat.org/title/wais-iii-escalaweschler-de-inteligencia-para-adultos-iii/oclc/54053545). This scale provided scores for a Full-Scale IQ (FSIQ), Verbal IQ (VIQ), and Performance IQ (PIQ) along with four secondary indices: PO, PS, VC, and WM. The subtests included in each index were as follows: PO: picture completion, block design, matrix reasoning; PS: digit-symbol coding and symbol search; VC: vocabulary, similarities, information, comprehension; and WM: arithmetic, digit span, letter-number sequencing.

The intelligence raw measures were scored according to the official normative data included in the printed version of WAIS-III. However, to avoid cultural bias, they were subsequently standardized with information from the Cuban sample to produce scores of the specific performance, adjusted for age for our population. The results included subtests, the verbal and performance subscales and full scale IQ for all the subjects, available in the ***WAIS_III.csv*** file. This dataset was employed in one study about how the white matter (FA-tracts based) predicts fluid and crystallized intelligence^20^.

#### Go No-Go test

For a subset of 56 participants, reaction times were recorded using a Go No-Go paradigm which consisted in a visual attention task, implemented using the psychophysiology software for cognitive stimulation Mindtracer^21^ (N_P-SW 1.3 v.2.1.0.0 Neuronic S.A.).

**Fig. 2:**
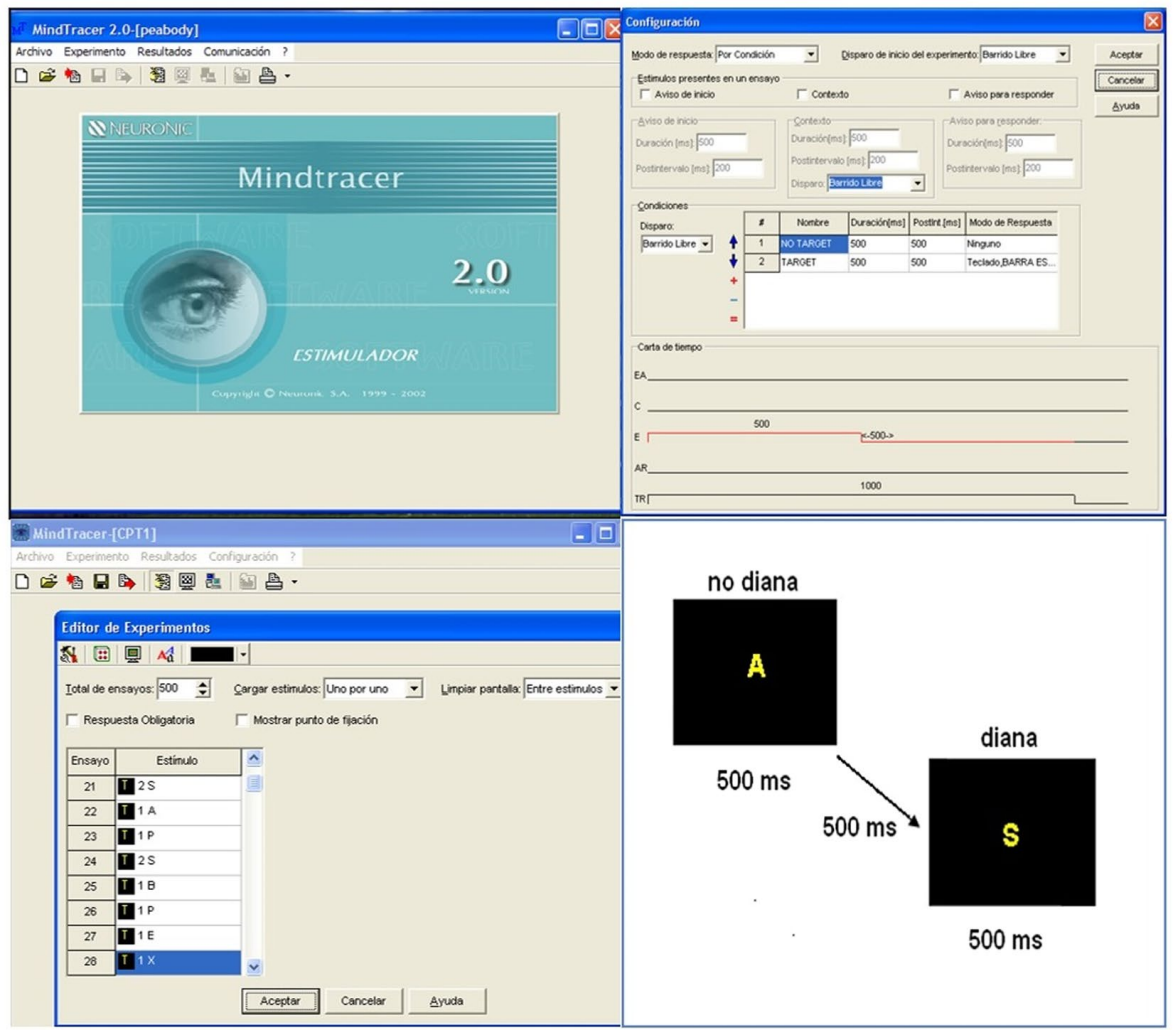
Top left: The main display of the software MINDTRACER for the preparation and presentation of the stimuli of the reaction time task. Top right.- Software options to design the experimental task. Bottom left.- Description of the different trials. Bottom right: Design of the presentation of the complex stimuli.

The task consisted of 500 trials, 25% in GO condition and 75% in NO-GO condition, where the stimuli was a set of letters: P B X E A S. The instructions for the participants were: Simple Reaction Time (RT1) consign: “Press the spacebar when “S” appears at the screen”. Complex Reaction time (RT2) consign: “Press the spacebar only when the letter “S” appears preceded by the letter “A”. The two tasks were presented to all the participants in the same consecutive order. For a description of the facilities of the software see Fig. 2. The results included in the file ***Reaction_Time.csv*** are: mean, standard deviation and skewness of the simple and complex reaction time tasks in milliseconds.

### EEG recordings

Resting-state EEG was recorded using the digital electroencephalograph system MEDICID 5-with 64 and 128 electrodes with differential amplifiers and gain of 10,000. The amplifiers used three filters: 1) Low cutoff (-3dB, high-pass): first order (6 dB/octave) 2) High Cutoff (-3dB, low-pass): Butterworth, second order (12 dB/octave) and 3) Line filter with a unit frequency response.

Electrodes were placed according to the 10–10 International System with a customized electrode cap. Linked earlobes were used as the EEG reference. Electrode impedances were considered acceptable if less than 5 KΩ. The bandpass filter parameters were 0.5–50 Hz and 60 Hz notch, and a sampling period of 200 Hz. The EEG was recorded in a temperature and noise-controlled room while the participant was sitting in a reclined chair. All individuals were asked to relax and remain at rest during the test to minimize artifacts produced by movements and to avoid excessive blinking. The participants received instructions to have enough sleep the previous night, take breakfast, and wash the hair before attending this appointment. See Table 7 for a summary of the technical parameters of the EEG. The structure of raw EEG recording was generated in the default format of the MEDICID neurometrics system (*.plg extension), which later is converted to standard BIDS format^22^. See the data records section.

**Table 7:** EEG equipment & recordings technical parameters.

Equipment	MEDICID 5
Number of channels	64/120
Gain	20,000/1000
Impedances	<5 KΩ.
Line Frequency	60 Hz
Amplifiers bandwidth	0.5 to 50 Hz
Sampling frequency	5msec./200 Hz.
Room Temperature	20 to 22° C.

### Electrode placement

**Fig. 3:**
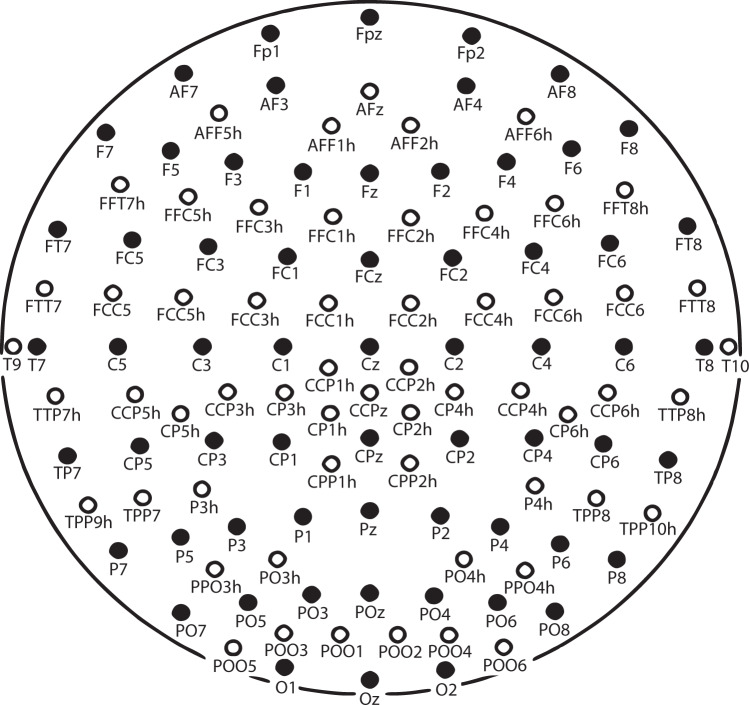
Schematic representation of the position of the electrode. In black the subset of electrodes employed in the 64 channels (maximum 62 cephalic electrodes). In white, the other electrodes were used for the 120 channels montage (maximum 120 cephalic electrodes). Both configurations use three additional channels which are employed to record the Electro-oculogram and Electrocardiogram.

Two different montages were employed, one with 64 channels and other with 120 channels as illustrated in Fig. 3 with different colors black (64) and white (120) to identify each montage. The nomenclature of the electrodes employed in the MEDICID system and their standardization is included in online-only Table 1 “Nomenclature of electrodes position.xlxs”

**Description of the EEG protocol comprising the following participant condition:**

1. Baseline: resting-state EEG with closed Eyes (state A), 10 minutes
2. Reactivity: this test consisted of the consecutive opening and closing eyes with an interval of 12 seconds. Open eyes (state B), 5 minutes, where the participant was instructed to look at a point, keeping the pupils fixed.
3. Hyperventilation (HPV): Dividing it in the first minute HPV1 (state C), the subject was instructed to start taking air through the nose and to breathe deeply. The second minute HPV2 (state D), and HPV3 (state E), this last one minute with more frequent and shallow inspirations. Total 3 minutes.
4. Recovery (state F): The last step is the recovery of the patient after the HPV, which lasted around one and a half minutes, but was recorded for 2 full minutes.

Note that the subject’s recordings were monitored continuously by the technician, to avoid contamination of the EEG with the electromyogram interference, other changes in the direct current level due to sweating, and also to prevent drowsiness. Any of these artifacts were annotated online by the technician.

Therefore, recordings of at least half an hour were ensured. A design requirement was to have enough valid EEG to carry subsequent frequency domain analysis. For this, quasi-stationary EEG epochs were selected, each consisting of 512-time samples, or 2.56 seconds being marked online continuous EEG recordings. Due to the high density of electrodes, the number of epochs for further analysis was guaranteed to be at least 50 windows for 64 channels and 80 windows for 120 channels (For details on analysis see^12^)

### MRI procedure

MRI: Magnetic resonance imaging (MRI) was performed on a 1.5 Tesla scanner (MAGNETOM Symphony Siemens Erlangen Germany). Over the course of MRI data acquisition, the scanner remained stable and did not undergo any major maintenance or updates which would systematically affect the quality of data provided here. The total measurement time was 45 minutes. See the MRI protocol used in Table 8.

**Table 8:** MRI Protocol.

Protocol	Description
T1w	• Patient position: First Supine Head • Sequence: 3D MPRAGE • FOV: 256 × 256 × 160 mm • Voxel dimensions: 1 × 1 × 1 mm • Inversion Time (TI): 1100 ms • Repetition Time (TR): 3000 ms • Echo Time (TE): 3.93 ms • Flip Angle: 15 degrees • Series Name: t13d_anatVOL • Name of the sequence: tfl3d1_ns brusque and remarkable.
Field Maps	• Sequence: 2D Double-echo gradient-echo field map • FOV: 224 × 224 mm • Imaging matrix: 64 × 64 • In-plane resolution: 3.5 × 3.5 mm • Number of slices: 40 • Slice orientation: Axial • Slice thickness: 3.5 mm • Slice gap: 0 mm • Repetition Time (TR): 672 ms • First Echo Time (TE1): 7.71 ms • Second Echo Time (TE2): 12.47 ms • Acquisitions: 1 • Flip Angle: 60 degrees • Image type: Magnitude and Phase • Name of the Series: fieldmap_pha or fieldmap_mag depending on the type • Name of the Sequence: fm2d2r.
Diffusion-weighted images (DWI) Variant 1	• Sequence: 2D Single-Shot Gradient Echo Echo-Planar-Imaging (GE-EPI) • Parallel acquisition: none • Multiband: none • FOV: 256 × 256 mm • Imaging matrix: 128 × 128 • Phase-Encoding direction: AP • In-plane resolution: 2 × 2 mm • Number of slices: 25 • Slice orientation: Axial • Slice thickness: 3 mm • Slice gap: 3 mm • Repetition Time (TR): 7000 ms • Echo Time (TE): 160 ms • Flip Angle: 90 degrees • Acquisitions: 6 • Diffusion gradient scheme: Siemens MDDW • Number of diffusion gradient directions: 12 • Echo Spacing: 1.149 ms • Readout Time: 145.92 ms • Image type: Magnitude • Series Name: ep2d_diff12dir • Name of the Sequences: ep_b0, ep_b1200 # 0… 11 This DWI sequence was repeated in a second run with the same parameters. The only difference was the position of the slices. They were translated parallel to the axis normal to the slice (axial plane) so that the single from the gaps of the first run were acquired in the second run. Consequently, the gaps of the second run occupied the regions from which the signal in the first run was acquired. In this way, the entire brain was covered with a total of 50 slices.
Diffusion-weighted images (DWI) Variant 2	• Sequence: 2D Single-Shot Gradient Echo Echo-Planar-Imaging (GE-EPI) • Parallel acquisition: none • Multiband: none • FOV: 256 × 256 mm • Imaging matrix: 128 × 128 • Phase-Encoding direction: AP • In-plane resolution: 2 × 2 mm • Number of slices: 25 • Slice orientation: Axial • Slice thickness: 3 mm • Slice gap: 0 mm • Repetition Time (TR): 7000 ms • Echo Time (TE): 160 ms • Flip Angle: 90 degrees • Acquisitions: 6 • Diffusion gradient scheme: Siemens MDDW • Number of diffusion gradient directions: 12 • Echo Spacing: 1.41 ms • Image type: Magnitude This sequence was not repeated.

Table 7 summarizes the technical specifications for the acquisition of the MRI. Note that only 203 participants have useful MRI images. Concerning diffusion images, we collected 201 participants with two variants: Variant 1 was performed on 148 subjects and Variant 2 on 53 subjects. The DWI specific parameters can be found in the metadata provided by BIDS for each participant. All DWIs were visually inspected and those which presented either technical or pathological defects were discarded.

Several EEG and MRI studies using this dataset has been published. One such study demonstrated how the cortical surface area could explain the morphological connectivity of brain networks^23^. Other studies explained the substantial inter-individual variability on the neuroanatomical determinants of EEG spectral properties using the DWI-fractional anisotropy^24^. Two papers studied the human brain anatomical network via diffusion-weighted MRI and Graph Theory characterizing brain anatomical connections^25,26^. A general framework for the tensor analysis of single-modality model inversion and multimodal data fusion was presented using our neuroimaging data as an example^27^.

## Data Records

BIDS (Brain Imaging Data Structure) is the new standard for the organization and description of the datasets containing neuroimaging (MRI, MEG, EEG, iEEG, NIRS, PET) and behavioral information^28^.

Based on this BIDS structure, we developed a methodology with the following steps:

1. Anonymization of EEG recordings and MRI scans.
2. Defacing of the MRI scans
3. Conversion of EEG recordings and MRI to BIDS-EEG
4. Validation of the BIDS structure

### Anonymization

We developed an application (*anomplg.exe*) to erase all the personal information stored in the EEG recordings in plg format, which could facilitate the identification of the participants. This application generated a secured copy of the personal information before its elimination.

The anonymization of the MRI neuroimages was performed using the script Dicat.py V 1.2 https://github.com/aces/DICAT developed by MCIN (McGill Center for Integrative Neuroscience) Montreal, Canada. All the data was anonymized with DICAT in 4 folders (ID, Patient_Name, Sex, Birth_Date).

### Defacing

The defacing process consisted in the elimination of the section with the face of the subject inside the anatomical MRI. This prevents the identification of the subject if a posterior 3D rendering is employed with the MRI scan. The software employed was the Mri_deface V 1.2, del FreeSurfer https://surfer.nmr.mgh.harvard.edu/fswiki/mri_deface.

### Conversion to BIDS

#### EEG

We developed an ad-hoc application (*plg2bids.exe*) to read the original EEG recordings in NEURONIC format and convert them into BIDS structure. This application is designed to read either individual EEG recordings or folders with multiple recordings and can update a current BIDS structure with new recordings.

#### MRI

The conversion of MRI neuroimages to BIDS structure was using the Dcm2Bids https://github.com/cbedetti/Dcm2Bids which generates the MRI BIDS structure with the original data in format DICOM.

The BIDS-EEG and MRI-BIDS structures were combined in only one structure using a software to combine EEG-MRI-BIDS (*joinbids.exe*).

### Validation

The final step was the validation of the BIDS structure using the web Bids-validator. https://bids-standard.github.io/bids-validator/

The dataset is hosted at Synapse.org, see^1^ 10.7303/syn22324937 and complete access is possible after login in the system. The dataset is also imported in the Longitudinal Online Research and Imaging System (LORIS) v20.2. https://mcin.ca/technology/loris/

## Technical Validation

For the quality control of the EEG, the MRI and psychological tests data of this study were implemented in a workflow:

### EEG

1. The ocular movements and other incidences were annotated by the technician during the recordings.
2. A Board of Certified clinical neurophysiologist reviewed the recorded raw EEG by visual inspection to provide:
  - Overall assessment of the EEG recordings quality to determine whether the recording should be repeated.
  - Scoring of semi-quantitative scales for abnormalities which could motivate the exclusion of the participant from the normative sample.
  - Selection of artifact-free EEG segments useful for further analysis in the continuous recordings.

### MRI

1. Automatic inspection was performed to check the protocol parameters of the MRI images using in-house software which generate a file with the value of the parameters.
2. Visual inspection by several clinical radiologists to detect abnormalities to decide if the participant should be excluded.
3. As part of the MRI quality control process, several MRI T1 images studies were fixed when a wrap-around artifact (without overlapping with the head) was detected by using the in-house Quality Control MRI app.

### Psychological tests and behavioral information

1. Supervision of the assessment sessions by one board-certified Clinical Neuropsychologist.
2. After input to the system, curation of the clinical, psychological, and demographical data was carried by a CNEURO team, assisted by statistical summary tools, to ensure quality control.

## Usage Notes

Each participant was assigned one ID with the structure **CBMxxxx** 3 characters identifying “Cuban Brain Mapping” and four digits indicating the number or order of the participant in the dataset. Note that the code is the same as the different data modalities of the subject. The dataset included BIDS files, the in-house programs, the psychological (WAIS-III, MMSE and reaction time), and the demographic and handedness data (∗.csv) available at Synapse.org. See reference^3^. You can visualize the data at 10.7303/syn22324937. To download them you need to be registered at the synapse.org website.

All the datasets have also been stored in the McGill Centre for Integrative Neuroscience (MCIN) network. The dataset will be available by request at https://chbmp-open.loris.ca

As mentioned before, due to an official request, there is a gender imbalance in the sample. This should be taken into consideration when doing statistical analysis.

While this data will provide greater ethnic and geographical diversity by providing a large multimodal dataset from a Latin-American setting, it is important to consider differences in EEG and MRI recording equipment. The interindividual variability of EEG characteristics seems to be much higher than country or equipment specific sources of variance^5,29–32^. However as mentioned in the section on EEG recording the characteristics of the EEG amplifiers used are provided to facilitate more detailed analyses of these factors. The issue of site and MRI scanner harmonization must be taken into consideration^33,34^.

We do note that this is a normative sample, selected to be functionally healthy with exclusion of any possible factor impacting brain health. The participants also had the benefit of an adequate life-long diet, free national educational and health system, most subjects with early stimulation programs during their infancy as provided by daycare centers. When comparing this data to other populations this is something to be taken into consideration. In an early multinational study^29^ the most important factor predicting deviations from EEG normality was socio economic status. The availability of this dataset can be useful in further studies of this type, now incorporating MRI information.

## Data Availability

The software developed by EAV is at https://github.com/eduardo-aubert/CHBMP-Code and 10.7303/syn22324937. The codes are: 1) Anomplg.exe: to anonimize the EEG records, deleting all the personal information stored in the EEG recordings, which could facilitate the identification of the participants. 2) Plg2bids.exe: to read the original EEG recordings in NEURONIC format and convert them to BIDS structure. 3) Joinbids.exe: to combine BIDS-EEG and MRI-BIDS into only one structure EEG-MRI-BIDS.

## Online-only Table

**Online-only Table 1:** Nomenclature of electrodes position.

MEDICID 5	Standard	MEDICID 5	Standard	MEDICID 5	Standard
1	Fp1	33	POO6	65	CPZ
2	Fp2	34	FT7	66	POZ
3	F3	35	FT8	67	OZ
4	F4	36	FFC3h	68	AFF1h
5	C3	37	FFC4h	69	AFF2h
6	C4	38	P5	70	FFC1h
7	P3	39	P6	71	FFC2h
8	P4	40	FC5	72	CCP1h
9	O1	41	FC6	73	CCP2h
10	O2	42	FCC5h	74	CP1h
11	F7	43	FCC6h	75	CP2h
12	F8	44	C5	76	CPP1h
13	T7	45	C6	77	CPP2h
14	T8	46	TTP7h	78	PO3
15	P7	47	TTP8h	79	PO4
16	P8	48	CPP5h	80	POO1
17	Fz	49	CPP6h	81	POO2
18	Cz	50	FTT7h	82	CP1
19	Pz	51	FTT8h	83	CP2
20	F1	52	TP7	84	AFF5h
21	F2	53	TP8	85	AFF6h
22	CP3h	54	PO5	86	FFC5h
23	CP4h	55	PO6	87	FFC6h
24	P1	56	T9	88	CCP3h
25	P2	57	T10	89	CCP4h
26	AF3	58	AF7	90	CP3
27	AF4	59	AF8	91	CP4
28	FCC1h	60	FFT7h	92	PPO5h
29	FCC2h	61	FFT8h	93	PPO6h
30	CPP3	62	FpZ	94	PPO3h
31	CPP4	63	AFz	95	PPO4h
32	POO5	64	FCZ	96	C1
97	C2	105	CP6h	113	CP6
98	F5	106	POO7h	114	PO7
99	F6	107	POO8h	115	PO8
100	FC3	108	FCC3h	116	FTT9h
101	FC4	109	FCC4h	117	FTT10h
102	FC1	110	CCP5h	118	TPP7h
103	FC2	111	CCP6h	119	TPP8h
104	CP5h	112	CP5	120	CCPz
